# Assimilation and turnover rates of lipid compounds in dominant Antarctic copepods fed with ^13^C-enriched diatoms

**DOI:** 10.1098/rstb.2019.0647

**Published:** 2020-06-15

**Authors:** Martin Graeve, Lauris Boissonnot, Barbara Niehoff, Wilhelm Hagen, Gerhard Kattner

**Affiliations:** 1 Ecological Chemistry Department, Alfred Wegener Institute Helmholtz Centre for Polar and Marine Research, 27570 Bremerhaven, Germany; 2 Marine Zoology, BreMarE Bremen Marine Ecology, University of Bremen, 28334 Bremen, Germany; 3 Aqua Kompetanse AS, R&D Department, N-7770 Flatanger, Norway

**Keywords:** Antarctic, zooplankton, lipids, carbon turnover, CSIA

## Abstract

The study revealed species- and stage-specific differences in lipid accumulation of the dominant Antarctic copepods, the primarily herbivorous *Calanoides acutus* (copepodite stage V (CV), females) and the more omnivorous *Calanus propinquus* (females) storing wax esters and triacylglycerols, respectively, which were collected in summer (end of December). Feeding carbon-labelled diatoms to these copepods, ^13^C elucidated assimilation and turnover rates of copepod total lipids as well as specific fatty acids and alcohols. The ^13^C incorporation was monitored by compound-specific stable isotope analysis (CSIA). CV stages of *C. acutus* exhibited an intense total lipid turnover and 55% of total lipids were labelled after 9 days of feeding. By contrast, total lipid assimilation of female *C. acutus* and *C. propinquus* was lower with 29% and 32%, respectively. The major dietary fatty acids 16:0, 16:1(n − 7) and 20:5(n − 3) had high turnover rates in all specimens. In *C. acutus* CV, the high rates of the de novo synthesized long-chain monounsaturated fatty acids and alcohols 20:1(n − 9) and 22:1(n − 11) indicate intense lipid deposition, whereas these rates were low in females. The differences in lipid assimilation and turnover clearly show that the copepod species exhibit a high variability and plasticity to adapt their lipid production to their various life phases.

This article is part of the theme issue ‘The next horizons for lipids as ‘trophic biomarkers': evidence and significance of consumer modification of dietary fatty acids'.

## 1. Introduction

In marine ecosystems, primary production varies considerably throughout the year, especially in boreal, polar and upwelling regions. To cope with unfavourable conditions, i.e. periods of low food supply, herbivorous zooplankton, in particular calanoid copepods, have developed specific biochemical strategies [1–3]. They massively accumulate and store neutral lipids during the productive season, usually in the form of wax esters or triacylglycerols. These lipid deposits are used as energy reserves for overwintering, e.g. during diapause, and for reproduction [4]. The large, lipid-storing copepods are an important component of the pelagic food web, and the accumulated and metabolized dietary lipids are transferred through the food web to higher trophic levels such as fish, birds and even whales [5].

The polar oceans especially are characterized by a short and intense period of primary production during summer and an extended period of food scarcity in winter due to light limitation and ice cover [1,6]. Here, phytoplankton blooms, usually dominated by diatoms, develop when illumination is increasing and ice is melting [7], providing a rich food source for herbivorous zooplankton. In the Antarctic, the calanoid copepods *Calanoides acutus* and *Calanus propinquus* are dominant components of the zooplankton community. Together, they may comprise more than half of the mesozooplankton biomass [8,9]. These two species have developed different life-cycle strategies. *C. acutus* is primarily herbivorous and survives the wintertime in a resting stage called diapause [4]. Its extremely large lipid store (greater than 50% of dry mass) consists of wax esters with characteristic long-chain fatty acids and alcohols typical of diapausing species [1,4]. Compared to *C. acutus*, *C. propinquus* has a broader diet, switching from a phytoplankton-based diet in summer to opportunistic feeding in winter. This is also reflected in its triacylglycerol storage, which is typical of species active year-round [1,9–11].

The huge lipid deposits accumulated by herbivorous zooplankton species, in particular calanoid copepods, are partly biosynthesized de novo from the phytoplankton diet, but they also contain specific phytoplankton fatty acids incorporated without biochemical alteration. The latter fatty acids are used as so-called trophic markers. Examples are 16:1(n − 7) typically abundant in diatoms and 18:4(n − 3) characteristic of flagellates [12,13]. The occurrence of these trophic markers indicates dietary preferences and reflects changes in feeding behaviour. Feeding experiments with the large Arctic *Calanus* species showed that it takes two to three weeks to fully change their diatom-dominated fatty acid pattern to a signature typical of flagellates, and vice versa [13,14]. Omnivorous species ingest a more diverse diet, which results in less specific lipid signatures [15,16].

Lipid biomarkers do not provide reliable rates of assimilation and turnover of specific fatty acids and alcohols. Using ^13^C-labelled phytoplankton diet and compound-specific isotope analyses of lipids, however, allows following the assimilation of dietary carbon into specific lipid compounds of copepods and assessing uptake rates. When wax ester-storing Arctic *Calanus* species were fed with labelled diatoms, their labelled fatty acids were rapidly taken up and fatty acids, and notably alcohols, were synthesized de novo to finally produce wax esters [14,17]. Recently, Boissonnot *et al*. used the same labelling technique on small Arctic copepods [18]. They showed that the herbivorous *Pseudocalanus minutus* exhibited high lipid turnover rates and that the species incorporated fatty acids more rapidly from diatoms than from flagellates. The lipid turnover rates in *Oithona similis* were lower, but this species was able to ingest and/or assimilate lipids with the same intensity from both food sources, possibly owing to its more flexible feeding behaviour and thus being better adapted to a broader dietary spectrum. These studies indicate that tackling assimilation dynamics of lipids using labelled food has the potential to better understand lipid synthesis processes and how they reflect life-history traits. Such knowledge is still completely lacking for Antarctic copepods.

For the first time, we therefore applied the compound-specific isotope analysis (CSIA) approach to elucidate the lipid dynamics of the dominant Antarctic copepods *C. acutus* and *C. propinquus* to assess the transfer and turnover rates of carbon into individual lipid compounds. We examined the hypothesis that wax esters can be more rapidly biosynthesized than triacylglycerols, since the reduction of fatty acids to alcohols diminishes a negative feedback mechanism, which occurs in the presence of surplus fatty acids [2]. It might also be possible that the biosynthetic production rate is similar for wax esters and triacylglycerols, but the wax ester synthesis via alcohol production is more efficient. In this respect, *C. acutus* and *C. propinquus* are ideal for comparing species with deviating lipid storage modes [9].

## 2. Material and methods

### (a) Sampling

Samples were taken during the Antarctic expedition PS 79 (ANT XXVIII/2) with RV *Polarstern* (Cape Town – Cape Town, 3 Dec. 2011–5 Jan. 2012). Copepods were collected at Station 53 (60° 3.22′S, 0° 2.14′ E) in the Antarctic Weddell Gyre on 28 December 2011 by vertical bongo net hauls down to 300 m depth. Specimens of *C. acutus* (210 copepodids CV and 160 females) and of *C. propinquus* (125 females, no CV stages available) were gently sorted from the catch, maintained alive in filtered seawater at 0°C in a cooling container on board and transported to Germany at 0°C by airplane. The copepods arrived in excellent condition 9 days after the catch. Copepods are able to cope with starving conditions for longer periods [19]. The feeding experiments started immediately after arrival in a temperature-controlled room (0°C) at AWI in Bremerhaven, and copepod mortality was negligible.

### (b) Experimental setup

To provide the copepods with the labelled diet, the diatom *Conticribra weissflogii* [20] was cultured in filtered seawater (0.7 µm pore diameter) with f/2 Guillard medium. The diatoms were enriched with ^13^C by adding labelled sodium bicarbonate (NaH^13^CO_3_; 98% ^13^C) at a concentration of 15 mg l^−1^. The labelled bicarbonate was added regularly to ensure high ^13^C concentrations. Extra silicate was added to the medium. The cultures were kept at constant light and 4–6°C and stirred twice a day to keep cells suspended. Algae were grown for several days and maintained in the exponential growth phase. Samples for lipid analyses were taken by filtrating 300 ml duplicates of the algal cultures on GF/F filters (0.7 µm pore diameter).

Live and healthy-looking copepods of *C. acutus* (CV, females) and *C. propinquus* (females) were transferred in groups of 20–45 individuals each to 2 l glass bottles containing filtered seawater. Due to the limited number of specimens, only one sample for each group could be analysed per experimental day. To determine the starting conditions (day 0), samples with 17–32 specimens (CV, females) were immediately frozen at −80°C for lipid analyses. During the nine days of the experiment, copepods were kept at continuous light to imitate polar day conditions. Every three days one third of the seawater was exchanged with freshly labelled algal suspension (max. 154 357 cells l^−1^). The green colour of the incubation water indicated that copepods were fed *ad libitum* (greater than 200 µg C l^−1^). At day 1, 3, 6 and 9 of the experiment, all specimens of one bottle containing each species and stage (total of three bottles, *n* = 17–45) were collected and frozen at −80°C for lipid analyses.

### (c) Lipid analysis

Total lipid was extracted by homogenizing the copepods and the filters with algae in dichloro­methane:methanol (2:1 v:v), modified after Folch *et al.* [21]. As internal standard, tricosanoic acid methyl ester (23:0) was added to each sample. Transesterification of the lipid extracts was performed with 3% sulfuric acid in methanol for 4 h at 80°C under nitrogen atmosphere. Fatty acid methyl esters and fatty alcohols were determined using a gas chromatograph (HP 6890 N, Agilent Technologies Deutschland GmbH & Co. KG) [22]. Unknown peaks were identified by gas chromatography (GC)–mass spectrometry. The chromatograms were evaluated using the ChemStation software from Agilent. Total lipid mass per individual was calculated by summing up fatty acid and fatty alcohol masses.

### (d) Carbon isotope ratios

The ^13^C isotopic enrichment in fatty acids and alcohols was measured using a Thermo GC-c-IRMS system, equipped with a Trace GC Ultra gas chromatograph, a GC Isolink operated in combustion mode at 1000°C and a Delta V Plus isotope ratio mass spectrometer connected via a Conflo IV interface (Thermo Scientific Corporation, Bremen, Germany). Fatty acid methyl esters and alcohols were analysed in splitless mode and separated on a DB-FFAP column [22]. Isotopic ratios of each fatty acid and alcohol are normally expressed in *δ* notation, but for this study, we used the atom per cent values, calculated by the Isodat 3.0 software, which is more appropriate than relative values to express isotope data in terms of isotope concentrations. The ^13^C enrichment that resulted from the assimilation of labelled food, the atom per cent excess, the proportion of carbon assimilated in the copepod fatty acids and alcohols, and the carbon assimilation as mass (µg C_assi_ ind^−1^) was calculated after Boissonnot *et al.* [18]. The carbon mass was derived from the number of moles of each fatty acid and alcohol in the copepods. The molecular mass of each labelled fatty acid and alcohol was calibrated by its carbon atom percentage to incorporate the carbon mass variation according to the ^13^C/^12^C ratio [23].

### (e) Statistics

Statistical analyses were performed using the free software R 3.2.1 (team RDC, 2010). Normal distribution was tested with the Shapiro–Wilk test with regard to ^13^C assimilation into fatty acids and alcohols presented as mass and percentage. Three regression types were tested to reflect the temporal trends: linear and polynomial degree 2 and 3. The best fit (*R*^2^) was found for linear regression for most compounds (however, linearity is only justified for the experimental period of 9 days). One-way ANOVA followed by Tukey honest significant difference tests were performed on the slope of linear regressions.

## 3. Results

### (a) Fatty acid composition and ^13^C labelling in *Conticribra weissflogii*

Major fatty acids of the diatom *Conticribra weissflogii* were 16:0 (13% of total fatty acids), 16:1(n − 7) (12.3%), 16:3(n − 4) (19.5%) and 20:5(n − 3) (17.2%). When we exposed the diatoms to ^13^C-labelled bicarbonate prior to our feeding experiments, ^13^C uptake in the algae increased exponentially and reached on average 37.4 ± 9.0 atom% after four days; thereafter, ^13^C enrichment of the algae was slightly variable but remained high.

### (b) Total lipid content and carbon assimilation into total lipids

**Figure 1.**
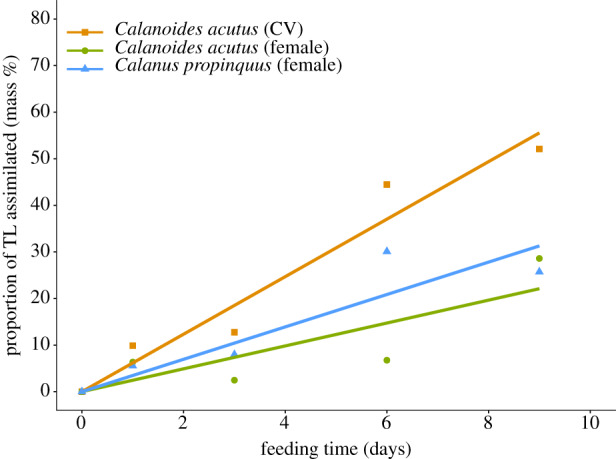
Assimilated lipids as proportion of total lipid mass (TL) of *Calanoides acutus* (CV stages, females) and *Calanus propinquus* (females) during the course of the experiment. The trend is illustrated by linear regression lines.

In *C. acutus*, the total lipid content (TL, sum of fatty acids and alcohols) was 15 ± 4.5 µg per CV and 63.8 ± 25 µg per female during the course of the experiment. Parallel analyses from the same station revealed similar low total lipid content of *C. acutus* CV (20 µg ind^−1^) comprising less than 10% of dry mass. ^13^C carbon assimilation was already detected after the first days of the feeding experiment. At the end of the experiment (day 9) (figure 1), lipid assimilation in *C. acutus* CV reached about 50% ^13^C_assi_ TL^−1^. This accounted for 5.8% day^−1^ for the nine days of the experiment. Females of *C. acutus* assimilated about 28% ^13^C_assi_ TL^−1^ into fatty acids. The lipid assimilation rate was 3.1% day^−1^.

*C. propinquus* females had a mean total lipid content of 34 ± 19 µg ind^−1^ which is also comparable to females at more southerly stations. They assimilated about 26% ^13^C_assi_ TL^−1^ into total lipids comparable to *C. acutus* females (figure 1). This is identical with the assimilation into fatty acids, because *C. propinquus* do not produce wax esters. The assimilation rate of fatty acids averaged 3.9% day^−1^.

### (c) Carbon assimilation into fatty acids and alcohols

#### (i) *Calanoides acutus* CV stage

At the start of the experiment, major fatty acids of *C. acutus* CV analysed by conventional GC-FID (table 1) were 22:6(n − 3), 20:5(n − 3), 18:1(n − 9) and 16:0 (in descending order), each contributing more than 10% of total fatty acids, followed by 18:2(n − 6) (8.9%) and several fatty acids accounting for less than 6%. During the course of the experiment, 20:5(n − 3) strongly increased (plus 12.8%) as well as 20:1(n − 9), 22:1(n − 11), and 16:1(n − 7). By contrast, all fatty acids with 18 carbon atoms and 22:6(n − 3) decreased. The alcohol 16:0 remained constant around 15%. The contribution of the 20:1(n − 9) alcohol strongly increased from 38.6% to 44.7%, whereas that of 22:1(n − 11) decreased from the initial 25.5% to 20.4% at the end of the experiment.

**Table 1.** *Calanoides acutus* (CV stages and females) and *Calanus propinquus* (females). Major fatty acids and alcohols (mass%). Changes in compositions from the start (day 0) to the end of the feeding experiment (day 9).

	*C. acutus* CV	*C. acutus* fem.	*C. propinquus* fem.
fatty acids	start–end	start–end	start–end
14:0	4.6–3.4	8.5–6.1	3.3–2.3
16:0	10.9–9.0	6.4–7.2	20.9–17.3
16:1(n − 7)	5.7–8.8	12.1–9.7	5.0–7.1
16:2(n − 4)	0.9–3.9	0.8–1.4	1.8–3.4
16:3(n − 4)	0.0–1.5	1.7–1.6	3.2–4.7
18:0	4.5–0.6	0.5–0.4	2.2–1.1
18:1(n − 7)	4.0–1.4	1.6–1.4	2.1–2.0
18:1(n − 9)	12.3–1.8	6.4–4.2	1.1–1.3
18:2(n − 6)	8.9–1.7	2.5–1.7	0.5–1.2
18:4(n − 3)	0.7–1.4	0.7–0.4	0.2–0.5
20:1(n − 9)	2.9–10.4	10.0–20.9	0.1–0.2
20:5(n − 3)	12.7–25.5	10.5–12.1	31.0–27.7
22:1(n − 11)	1.9–5.3	15.5–11.9	0.6–0.1
22:1(n − 9)	0.9–1.5	4.7–3.6	0.5–0.2
22:6(n − 3)	14.1–10.4	10.2–5.8	16.4–17.3
24:1(n − 9)	3.1–5.7	0.4–4.4	3.5–8.3
alcohols			
14:0	9.2–6.1	5.2–3.9	
16:0	15.3–14.6	6.2–4.8	
16:1(n − 7)	4.0–5.3	1.5–1.5	
18:1(n − 9)	4.8–1.4	1.0–0.3	
18:1(n − 7)	2.6–0.9	0.4–0.3	
20:1(n − 9)	38.6–44.7	53.4–53.4	
22:1(n − 11)	25.5–20.4	32.3–29.3	

**Figure 2.**
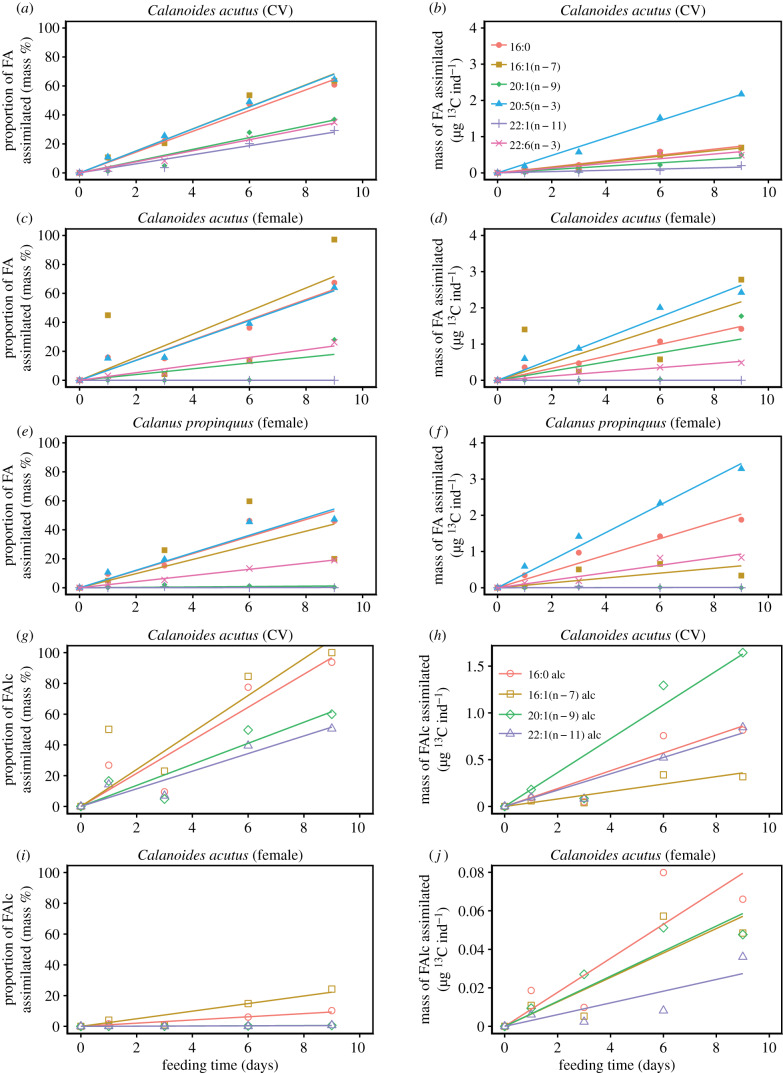
Proportion of carbon assimilation (mass% carbon) and mass of carbon assimilation (µg C ind^−1^) into major FA (*a–f*) and FAlc (*g–j*) of *Calanoides acutus* (CV, females) and *Calanus propinquus* (females) during the course of the experiment. Statistical data are presented in electronic supplementary material, table S1 and S2.

The assimilation of carbon into all lipid compounds of *C. acutus* CV was significant (*p* < 0.01; electronic supplementary material, table S1 and S2). The fatty acid 20:5(n − 3) showed maximum carbon assimilation with 2.2 µg C_assi_ ind^−1^ by day 9. By contrast, carbon assimilation into most other fatty acids, especially 18:1(n − 7) and 22:1(n − 11), was relatively low with less than 0.7 µg C_assi_ ind^−1^ during the entire experiment. Carbon uptake into the fatty alcohols of *C. acutus* CV ranged from 0.3 to 1.6 µg C_assi_ ind^−1^ with maximum uptake for 20:1(n − 9). Carbon assimilation into alcohols essentially started after day 3 (figure 2).

On a percentage basis (assimilated fatty acids of fatty acid mass) the fatty acids 16:1(n − 7), 20:5(n − 3) and 16:0 showed the highest portions of assimilated carbon with more than 60%, which correspond to a mean daily assimilation of 10.4, 11.1 and 7.2% ^13^C_assi_ day^−1^, respectively (electronic supplementary material, table S3). Assimilated carbon of the other fatty acids 14:0, 22:6(n − 3), 20:1(n − 9) and 22:1(n − 11) accounted for 25–35%. The polyunsaturated 16:3(n − 4) comprising almost 20% of the diatom fatty acids as well as 16:2(n − 4) showed nearly 100% assimilation, but increased only slightly in the copepods. The prevailing alcohols were also highly ^13^C enriched. After a lag phase of three days, the 16:0 alcohol was labelled up to 90% (10.4% ^13^C_assi_ day^−1^) and the alcohols 14:0, 20:1(n − 9) and 22:1(n − 11) up to 50–60% at the end of the experiment.

#### (ii) *Calanoides acutus* females

In *C. acutus* females, the fatty acids 22:1(n − 11), 16:1(n − 7), 20:5(n − 3) and 22:6(n − 3) dominated with a portion greater than 10%, followed by 20:1(n − 9), 16:0, and 18:1(n − 9). Especially, 20:1(n − 9) showed a strong increase by 11.2%. The other fatty acids remained constant or decreased by 1.3% to 4.4%. The dominant alcohols 20:1(n − 9) and 22:1(n − 11) accounted for more than 80% of total fatty alcohols (table 1).

Total carbon assimilation into fatty acids of *C. acutus* females was slightly higher compared to the CV stages. The assimilation into 20:5(n − 3) and 16:0 was 3.5 and 1.4 µg C_assi_ ind^−1^, respectively. After a lag phase of six days, highest carbon incorporation occurred into 16:1(n − 7) (2.8 µg C_assi_ ind^−1^) and 20:1(n − 9) (1.8 µg C_assi_ ind^−1^). By contrast to the CV stages, the assimilation in fatty alcohols was clearly lower in the females ranging only from 0.04 to 0.07 µg C_assi_ ind^−1^ (figure 2).

Similar to the CV stages, the fatty acids 20:5(n − 3), 16:1(n − 7) and 16:0 were labelled with more than 60%, which corresponds to a rate of 10.8, 7.5 and 7.1% ^13^C_assi_ day^−1^ (electronic supplementary material, table S3). Labelling of the other fatty acids 14:0, 22:6(n − 3), 20:1(n − 9) and 22:1(n − 11) ranged between 15% and 25%. The major difference between CV and females was the weak ^13^C assimilation into the alcohols with only up to 10% of ^13^C.

#### (iii) *Calanus propinquus* females

Portions of major fatty acids of *C. propinquus* females remained rather constant throughout the experiment with maximum changes of only 5%. Principal fatty acids were 20:5(n − 3) (approx. 30%), followed by 16:0 and 22:6(n − 3) (approx. 20%). Contributions of 24:1(n − 9) and 16:1(n − 7) as well as 16:3(n − 4) and 16:2(n − 4) were clearly lower (table 1). Since triacylglycerols are the principal storage lipids in *C. propinquus*, no fatty alcohols were detected.

In *C. propinquus*, carbon assimilation into individual fatty acids was slightly higher compared to *C. acutus* females and CVs. For the non-algal fatty acids, 20:1(n − 9) and 22:1(n − 11) incorporation remained below 0.5 µg C_assi_ ind^−1^. Significant changes during the course of the experiment were only detected for the fatty acids 16:0 (*p* < 0.01), 20:5(n − 3) (*p* < 0.01) and 22:6(n − 3) (*p* < 0.001; electronic supplementary material, table S2), reaching 2.0, 3.5 and 1.0 µg C_assi_ ind^−1^, respectively, after 9 days (figure 2).

The percentage of assimilated fatty acids was about 50% for 16:0 and 20:5(n − 3) and 20% for 22:6(n − 3). The value for 16:1(n − 7) was also about 50% at day 6, but it decreased towards the end of the experiment. The daily assimilation rates of 16:0, 16:1(n − 7) and 20:5(n − 3) were 5.1, 2.2 and 5.3% ^13^C_assi_ day^−1^, respectively (electronic supplementary material, table S3). The long-chain monounsaturated fatty acids showed only minor labelling.

## 4. Discussion

The copepods used in our experiment were lipid-poor, although they had been collected in late December (summer), when phytoplankton food should still be available. Specimens can have very different amounts of storage lipid varying between 12% and 50% of total lipid per dry mass [24]. Possibly, the feeding conditions at our sampling station were not optimal for the two species, as indicated by low chlorophyll *a* concentrations measured by a CTD mounted fluorometer. However, low lipid levels are not unusual in late December, especially for *C. propinquus*, since females may have invested dietary energy primarily in egg production instead of accumulating storage lipids. Analyses of lipid-poor winter specimens revealed that females of *C. propinquus* totally lacked 20:1 and 22:1 fatty acids. In *C. acutus*, these storage fatty acids also occurred only at lower levels, similar to our data. By contrast, lipid-rich *C*. *acutus and C. propinquus* contain high amounts of long-chain monounsaturated moieties with chain lengths ≥20 carbon atoms, which comprise 50% and even more of total fatty acids and alcohols in the wax ester and triacylglycerol fractions, respectively [25,26].

The initial fatty acid patterns of both copepod species reflected a typical diatom-dominated diet in the field. Since diatoms were also offered during the feeding experiment, a considerable change in the relative fatty acid composition was not expected. Phytoplankton was provided in excess and therefore copepods were not food-limited [18]. They assimilated dietary lipids as indicated by the increase in labelled lipid compounds. Especially, 16:0, 20:5(n − 3) and 22:6(n − 3) fatty acids were taken up from the phytoplankton diet, which are dominant components in phospholipids and thus indispensable for biomembranes [2]. It is likely that especially 20:5(n − 3) also occurred in high amounts in the wax esters of *C. acutus,* as shown by Albers *et al*. [26], which suggests that the excellent feeding conditions during the experiment increased wax ester biosynthesis.

In the CV stages of *C. acutus*, a considerable fraction of the 20:1(n − 9) fatty acid was converted to the corresponding alcohol. ^13^C labelling revealed that almost all carbon of this alcohol was replaced by dietary carbon. Correspondingly, the concentration of the 20:1(n − 9) fatty acid increased only slightly. There seems to be a delay of about 3 days in the production of all alcohols. This may be due to the fact that the precursor fatty acids have to be produced in advance, mainly de novo, and then reduced to alcohols, which most likely requires a higher energetic effort. The pronounced biosynthesis of alcohols by *C. acutus* CV stages during feeding also shows that they were actively accumulating lipid stores by biosynthesizing wax esters.

By contrast, in the females of *C. acutus*, the biosynthesis of alcohols was generally low, although they still ingested dietary fatty acids and produced the 20:1(n − 9) fatty acid de novo after a lag phase of about 6 days. Although the portion of 20:1(n − 9) doubled in the fatty acid composition, the females did not convert this fatty acid to the corresponding alcohol, which is the major alcohol in this species [27]. This was clearly different compared to the CV stages and shows that there was no indication of an intense wax ester biosynthesis in female *C. acutus*. It is, however, possible that the females used the dietary fatty acids for gonad development and the production of eggs, but this has not been monitored. In the northern *Calanus* species, *C. glacialis* and *C. finmarchicus*, high egg production is related to food supply and egg-laying usually starts after several days of feeding. The eggs of these copepods contain only small amounts of wax esters and therefore wax esters may not be essential for egg production, besides offering energy [18]. However, nothing is known about the lipid composition of eggs from Antarctic copepods and it remains open whether the dietary fatty acids assimilated during our experiment were allocated to reproductive processes.

Polyunsaturated fatty acids with 16 carbon atoms, which belong to the major dietary fatty acids, were highly labelled in the copepods, but increased only slightly in mass. These fatty acids are not essential for membranes and our data suggest that they are not retained in storage lipids. However, in similar experiments with Arctic *Calanus* species, these fatty acids with 16 carbon atoms were clearly enriched during feeding [14]. Although this is not fully understood, copepods apparently store specific dietary fatty acids as energy reserves whereas they directly utilize others for energetic requirements.

Our results suggest that copepods, in particular lipid-poor specimens, start lipid accumulation with the uptake of fatty acids originating from the phytoplankton diet when food becomes available. After 3–6 days, the production of de novo synthesized lipids from proteins and carbohydrates as well as fatty acid precursors strongly increased. Potential fatty acid precursors are 14:0, 16:0 and 18:1(n − 9), although the latter only occurred in low amounts in the diatom (3.2%). The 16:1(n − 7) fatty acid, a major fatty acid of diatoms, is not elongated to long-chain monounsaturated fatty acids with 20 and 22 carbon atoms, because it would result in fatty acids with a double bond in n − 7 position [2,27]. Such fatty acids were only found in trace amounts in the copepods of our study and are generally minor components in calanoid copepods [26]. Our data show that fatty acid accumulation, directly assimilated and de novo synthesized, was similar in both species, females and CV stages. However, the intense alcohol assimilation and labelling of *C. acutus* CV suggest that wax ester deposition is advantageous, perhaps not faster but more efficient compared to storing triacylglycerols. Unfortunately, a direct comparison with *C. propinquus* CV stages was not possible.

When feeding ^13^C-labelled phytoplankton to zooplankton, it has to be considered that only a small part, usually less than 10%, of dry mass of the phytoplankton diet consists of lipids, while the main portion comprises proteins with few carbohydrates. Therefore, only a minor part of the food is directly transferred as phytoplankton lipids to zooplankton. Hence, the majority of the labelled dietary carbon is used for de novo production and energetic requirements. Part of the ingested carbon is also lost via respiration and egestion. Therefore, future studies should include the analysis of faecal pellets, although this may be challenging.

## 5. Summary and conclusion

The capacity of *C. acutus* and *C. propinquus* to assimilate dietary carbon in variable amounts into different lipid compounds depends on their developmental stages and their life-cycle strategies. In our study, CV stages of *C*. *acutus* were lipid-poor. This does not reflect the usual situation at the end of the primary production period in late summer, when the CV stages are already prepared for overwintering, including a wax ester-rich oil sac. In our experiment, the lipid-poor CV stages of *C. acutus* assimilated carbon at high rates. They produced fatty acids and alcohols, hence wax esters, indicating that they were able to continue lipid biosynthesis when food became available. By contrast, the females of both species had a down-regulated storage lipid metabolism with weak carbon assimilation into their storage lipids. At this stage of their life cycle, females seem to preferentially assimilate dietary fatty acids, which is less energy-consuming than de novo synthesis, but important for maintaining membrane activity. The differences in carbon and lipid assimilation clearly show that copepods exhibit a high variability and plasticity to adjust their lipid metabolism to the various life phases, which depend on the species, stage and on spatiotemporal conditions.

## Supplementary Material

Supplementary material-Graeve et al.

## Data accessibility

Additional primary data is available in the data repository PANGAEA: Graeve, Martin; Boissonnot, Lauris; Niehoff, Barbara; Hagen, Wilhelm; Kattner, Gerhard (2020): Assimilation rates, compound-specific stable isotope analysis and fatty acid data of dominant Antarctic copepods. PANGAEA, https://doi.org/10.1594/PANGAEA.913800. Further data related material is available as electronic supplementary material, S1–S3.
