# Supplementary material for: Assimilation and turnover rates of lipid compounds in dominant Antarctic copepods fed with 13C-enriched diatoms
Source: Philos Trans R Soc Lond B Biol Sci. 2020 Jun 15;375(1804):20190647. doi: 10.1098/rstb.2019.0647 (PMC7333956; doi:10.1098/rstb.2019.0647)
Supplement: Supplementary material-Graeve et al. [file rstb20190647supp1.docx]

**Supplementary material-Graeve et al.**

**Antarctic copepods: statistical data and turnover rates**

Table S1. Results of Fisher test (1-way ANOVA) - Percent fatty acid and alcohols assimilated from day 0 to day 9

| Compound | *C. acutu*s CV | |  | *C. acutu*s fem. | |  | *C. propinquus* fem. | |
| --- | --- | --- | --- | --- | --- | --- | --- | --- |
|  | R^2^ | p-value |  | R^2^ | p-value |  | R^2^ | p-value |
|  |  |  |  |  |  |  |  |  |
| Fatty acids |  |  |  |  |  |  |  |  |
|  |  |  |  |  |  |  |  |  |
| 16:0 | 0.98 | < 0.001 |  | 0.93 | < 0.01 |  | 0.97 | < 0.05 |
| 16:1(n-7) | 0.96 | < 0.01 |  | 0.35 | - |  | 0.11 | - |
| 20:1(n-9) | 0.94 | < 0.01 |  | 0.46 | - |  | 0.13 | - |
| 20:5(n-3) | 0.99 | < 0.001 |  | 0.95 | < 0.01 |  | 0.90 | < 0.01 |
| 22:1(n-11) | 0.95 | < 0.01 |  | 0.17 | - |  | 0.23 | - |
| 22:6(n-3) | 0.99 | < 0.001 |  | 0.93 | < 0.001 |  | 0.99 | < 0.001 |
|  |  |  |  |  |  |  |  |  |
| Alcohols |  |  |  |  |  |  |  |  |
| 16:0 | 0.82 | < 0.05 |  | 0.87 | < 0.05 |  |  |  |
| 16:1(n-7) | 0.72 | < 0.05 |  | 0.86 | < 0.05 |  |  |  |
| 20:1(n-9) | 0.81 | < 0.05 |  | 0.92 | < 0.01 |  |  |  |
| 22:1(n-11) | 0.86 | < 0.05 |  | 0.55 | - |  |  |  |

Table S2. Results of Fisher test (1-way ANOVA) - Mass of fatty acids and alcohols assimilated from day 0 to day 9.

| Compound | *C. acutu*s CV | |  | *C. acutu*s fem. | |  | *C. propinquus* fem. | |
| --- | --- | --- | --- | --- | --- | --- | --- | --- |
|  | R^2^ | p-value |  | R^2^ | p-value |  | R^2^ | p-value |
|  |  |  |  |  |  |  |  |  |
| Fatty acids |  |  |  |  |  |  |  |  |
|  |  |  |  |  |  |  |  |  |
| 16:0 | 0.95 | < 0.01 |  | 0.96 | < 0.01 |  | 0.95 | < 0.01 |
| 16:1(n-7) | 0.98 | < 0.01 |  | 0.31 | - |  | 0.19 | - |
| 20:1(n-9) | 0.89 | < 0.05 |  | 0.50 | - |  | 0.33 | - |
| 20:5(n-3) | 0.99 | < 0.001 |  | 0.95 | < 0.01 |  | 0.99 | < 0.001 |
| 22:1(n-11) | 0.83 | < 0.05 |  | 0.25 | - |  | 0.29 | - |
| 22:6(n-3) | 0.82 | < 0.05 |  | 0.94 | < 0.01 |  | 0.87 | < 0.05 |
|  |  |  |  |  |  |  |  |  |
| Alcohols |  |  |  |  |  |  |  |  |
| 16:0 | 0.82 | < 0.05 |  | 0.67 | - |  |  |  |
| 16:1(n-7) | 0.77 | < 0.05 |  | 0.70 | - |  |  |  |
| 20:1(n-9) | 0.87 | < 0.05 |  | 0.83 | < 0.05 |  |  |  |
| 22:1(n-11) | 0.92 | < 0.01 |  | 0.66 | - |  |  |  |
|  |  |  |  |  |  |  |  |  |

Table S3. Carbon assimilation rates per day calculated from data at the end of the experiment.

| Compound | Rates (%_assi_day^-1^) | | |
| --- | --- | --- | --- |
| Fatty acids | *C. acutus* CV | *C. acutus* fem. | *C. propinquus* fem. |
| 16:0 | 10.4 | 7.5 | 5.1 |
| 16:1(n-7) | 11.1 | 10.8 | 2.2 |
| 20:1(n-9) | 4.1 | 3.1 | 0.0 |
| 20:5(n-3) | 7.2 | 7.1 | 5.3 |
| 22:1(n-11) | 3.3 | 0.1 | 0.2 |
| 22:6(n-3) | 3.9 | 2.9 | 2.1 |
|  |  |  |  |
| Alcohols |  |  |  |
| 14:0 | 7.0 | 0.6 | - |
| 16:0 | 10.4 | 1.1 | - |
| 16:1(n-7) | 11.1 | 2.7 | - |
| 20:1(n-9) | 6.7 | 0.1 | - |
| 22:1(n-11) | 5.6 | 0.1 | - |
